# The adoption impact of wheat-chickpea double cropping on yield and farm income of smallholder farmers in Central Highlands of Ethiopia: the case of Becho district

**DOI:** 10.1016/j.heliyon.2021.e07203

**Published:** 2021-06-02

**Authors:** Desalegn Haileyesus, Abate Mekuriaw

**Affiliations:** a CASCAPE-BENEFIT Partnership, Addis Ababa, Ethiopia; b College of Development Studies, Addis Ababa University, Addis Ababa, Ethiopia

**Keywords:** Wheat, Chickpea, Adoption, Double cropping, Impacts, Yield, Farm income

## Abstract

This paper examines impacts of adoption of wheat chickpea double cropping on yield and farm income of smallholder rural farmers in Becho district, South West Shewa Zone, Oromia Region, Ethiopia. The study used cross-sectional data collected from 203 smallholder farm households selected randomly through two-stage stratified random sampling technique. Propensity score matching was employed to analyze the impacts of adoption on yield and farm income. The result showed that adoption of wheat-chickpea double cropping has significant impact on yield and farm income of the group of adopter households compared to the group of non-adopters. With regard to yield, adopters harvested average wheat yield of 2120 kg/ha, while the non-adopters harvested average wheat yield of 1420 kg/ha. In addition, the treated households earned average annual farm income of about 709.125 Euro per year from sale of both wheat and chickpea as adopters; while the non-adopters earned average farm income of 129 Euro from sale of wheat. These results imply that scaling out of wheat-chickpea double cropping contributes to food security and rural livelihood improvement through yield and farm income increment. Hence, encouraging farmers towards adoption of wheat-chickpea double cropping is essential for improving livelihoods of rural households by properly addressing factors such as access to improved seeds, training on double cropping, involvement in non-farm income activities, access to broad bed maker (BBM), ownership of tropical livestock unit (TLU) and access to fertilizer.

## 1 Introduction

Globally, multiple cropping systems such as intercropping, double or sequential cropping, mixed cropping and relay cropping have been in practice for environmental, productivity, efficiency and disease concerns. These cropping systems are asserted to improve soil health, reduce pest and disease incidence, enhance productivity and minimize risks associated with monocropping (Azam-Ali, 2003; Agegnehu et al., 2008; Adarsh et al., 2019; Waha et al., 2020).

Various combinations of double cropping are practiced in the world. Winter wheat followed by soybean is the most popular double cropping system in Missouri and much of the Midwest and Southern United States (Kyei-Boahen and Zhang, 2006). Wheat, a predominant winter crop in the Argentinean Pampas, is cultivated in sequential double cropping with soybean. Similarly, double cropping is practiced in Brazilain agriculture with maize, peanuts, potatoes and beans (SAGPYA, 2011 in Opstal et al., 2011; Elobeid et al., 2019). Following the catastrophic famine years of 1995 and 1996, North Korea adopted double cropping program in 1997, and the system became an established form of cropping system which helped to increase food grain while substantially reducing the need for food aid (FAO, 2003). Generally, multiple cropping (harvesting more than once a year) is a widespread land management strategy in tropical and subtropical agriculture and the majority of multiple cropping areas are found in East Asia and South Asia (Waha et al., 2020). According to Waha et al. (2020) estimation, 12% (135 million hectares) of global cropland is growing multiple crops (two or three crops in sequences).

Of the multiple cropping systems, pulse-cereal based double cropping is the most common form of crop combination practiced worldwide. Double cropping is a production system that involves the growth of two separate crops (usually cereals with pluses) at different times in the same growing season, whereby harvesting of one species is followed by the planting of another (Sandler, 2014). Pulses play an important role in the cropping system through high carbon sequestration, low carbon footprint, fixing atmospheric nitrogen in soils, low water footprint, hydrogen fertilization of soils, and improving soil biodiversity (Adarsh et al., 2019). Even Adarsh et al. (2019) stressed that pulse included cropping systems are the only way to enhance production as available farmland is becoming limited. Through double cropping, annual agricultural output could be enhanced without farmland expansion, and thus helps to enhance food security among smallholder farmers in particular and national economic growth in general; and it is particularly essential for places and countries where land is getting fragmented as a response to population increment, and Ethiopia is a typical example for this.

Agriculture is the dominant economic sector of Ethiopian economy which contributes a lion's share to the Gross Domestic Product, employment and foreign exchange earnings (CSA, 2016). Smallholder producers, which are about 12 million households, account for about 95% of agricultural GDP (CSA, 2016). Despite its importance, the sector is constrained by a number of biophysical and man-made predicaments. Besides, low input usage and shortage of agroecology specific technologies are hampering crop productivity and this continued to cause food insecurity among millions of people in the country. Population of Ethiopia has been increasing rapidly over the past four decades, from 35 million in the 1980s to 99.4 million in 2015 and passed 100 million in 2017. Farmland is getting fragmented from time to time, as rural population is increasing over years (Minot et al., 2015). The country has been a net importer of food for decades in its struggle to feed its growing population. Double cropping, wherever possible, helps to sustainably increase productivity and achieve food security (Jemberu et al., 2018).

Double cropping maximizes benefit from same area and season by enabling cultivation twice in a single season. It reduces the risk of field loss due to drought, insect and disease, and enhances a better use of vertical space and time in limited farmland. Its practice allows better distribution of water and nutrients through the soil profile, which in turn, contributes to increase yield. Double cropping of cereals with pulses has also an implication in nutrition security. For instance, as chickpea is rich in protein, double cropping with wheat contributes in alleviating malnutrition and improving human health (Eshete et al., 2017). In the era of continuous land fragmentation, double cropping helps to intensify yield vertically and thus should be considered as a package option for promotion (Beuerlein, 2001; Sandler, 2014; Jemberu et al., 2018). Given these important aspects of double cropping, farmers in different parts of the world grow cereals and pulses sequentially in the same land in the same season, and such practice provides farmers with an opportunity to increase production and farm income. The key question in such practice is to identify best combination and compatibility of both crops to exhaust the opportunity from the double cropping system (Beuerlein, 2001).

Wheat and chickpea are very important food crops in Ethiopian diet. Although the country is the second largest producer of wheat in sub-Saharan Africa, it could not yet meet the national demand, and large amount is being imported from abroad. The national mean wheat grain yield is still low (2.74 t/ha) (CSA, 2018). Boosting the crop's productivity through double cropping with important pulse crops such as chickpea could be a worthy option to contribute to food and nutrition security while improving soil fertility (Minta et al., 2014; CASCAPE, 2015). Chickpea is a high value legume crop in Ethiopia which supports the livelihoods of millions of smallholder farmers (Ferede et al., 2018) and the country is one of the world's largest producers and exporters of the crop (Bekele and Teklewold, 2007; Kassie et al., 2009; GAIN, 2018). Being the seventh largest producer in the world, the country accounts to 90 percent of the sub-Saharan chickpea production (Kassie et al., 2009). Since the crop is grown in rotation with cereals (mainly tef and wheat), it does not compete for land (Verkaart et al., 2017). Despite its importance to the country's economy and livelihoods of farmers, the productivity of the crop is low at 2.06 t/ha (CSA, 2018).

Ethiopia has a huge potential for double cropping practice in its Vertisol dominant areas such as West Shewa zone, South West Shewa zone, North Shewa Zone of Oromia region and West Gojjam, East Gojjam, North Gondar and South Gondar Zones of Amhara region (MoA, 2014). Although there is a wide opportunity, the practice of wheat-chickpea double cropping is minimal in the country (Asaduzzamam et al., 1989).

In the study area, Becho district, the practice of double cropping is getting better due to agricultural intervention carried out by CASCAPE^1^ project in the past 8 years. Through participatory evaluation of different improved wheat technologies, CASCAPE identified a bread wheat variety, known as *Hidase,* as a compatible variety to practice double cropping in the district and other areas with similar agroecologies. The variety matures averagely within 100–110 days in the main rainy season (June–September); leaving adequate growing period for the following crop (chickpea). This innovation helped farmers to harvest more produce within one season. As a result, the practice of double cropping of wheat-chickpea is increasing in the district. However, no study was conducted on the adoption of double cropping and its impact on productivity and income of farmers in the study area.

Available studies at international level, on the other hand, show mixed results on the same issue. For instance, Crabtree et al. (1990) reported that grain yield of wheat is less in double-cropped systems (2510 kg/ha when cropped with soybean) as compared to mono-crop systems (average of 3050 kg/ha). Similarly Crabtree and Rupp (1980) reported that double-cropped wheat yielded 2,210 kg/ha compared to 2,530 kg/ha for mono-cropped wheat. Caviglia et al. (2011), on the other hand, reported indifferent result, where double-crops (soybean with wheat) out yielded sole crops by 38–82% for soybean, but relative yield for wheat was not different both in sequential and relay intercropping. On the contrary, Marcellos et al. (1994) found out 40–50% wheat grain yield increment from double cropping as compared with wheat mono-cropped. Sandler (2014) also reported wheat having a three-fold yield advantage following soybean than grain yield in mono cropping, which was attributed to soil nitrogen fixing properties of the subsequent legume crop.

These results show that impact of double cropping on productivity has opposing results as double cropping is context and crop specific. In addition, multiple cropping systems are poorly accounted for in assessment of global food production and land use change although multiple cropping accounts to 12% of global cropland (Waha et al., 2020). On top of this, although a number of publications stress the importance of double cropping of wheat with pulses, these studies are more inclined towards to linking double cropping with yield, grain quality and soil fertility whereas largely ignoring the impact on income (Crabtree et al., 1990; Aslam et al., 2003; Caviglia et al., 2011). While these are research gaps, wheat-chickpea double cropping in Ethiopian context has also international effect as Ethiopia is Africa's top chickpea producer and exporter and the world's seventh largest producer. If the trend of wheat-chickpea double cropping expands, it will also have implication on the international market and foreign currency earnings for Ethiopia, and this warrants thorough understanding of the double cropping system on productivity and income. Against the backdrop of these gaps, this study, therefore, aimed to examine the impact of adopting wheat-chickpea double cropping on yield and farm income of smallholder farmers in Becho district, South West Shewa Zone, Oromia region, Ethiopia.

## 2 Materials and methods

### 2.1 Description of the study area

The study was conducted in the Central Highlands of Ethiopia taking Becho district as the study's destination. Becho district is located in the Southwest Shewa zone of Oromia region. The district is subdivided into 21 Kebeles (smallest administrative units in Ethiopia). From these, 19 Kebeles are rural and the other two are urban. The capital of the district, Tulu Bolo, is located at about 80 km southwest of Addis Ababa, the capital city of Ethiopia. The estimated population of the district in 2017 was 99, 090; among which 76.42% (75,724) were rural residents (CSA, 2013).

Geographically, the district is located between 8°34′59.99″ N and 38°14′60.00″ E, with an altitude ranging from 1,850 to 2,200 masl. Highlands, which roughly account 95% of the landmass, dominate the district. The remaining area is categorized under mid highland. The mean annual temperature of the district ranges from 16 to 25 °C and the mean annual rainfall is about 1,300 mm. The main rainy season extends from May to September.

**Figure 1:**
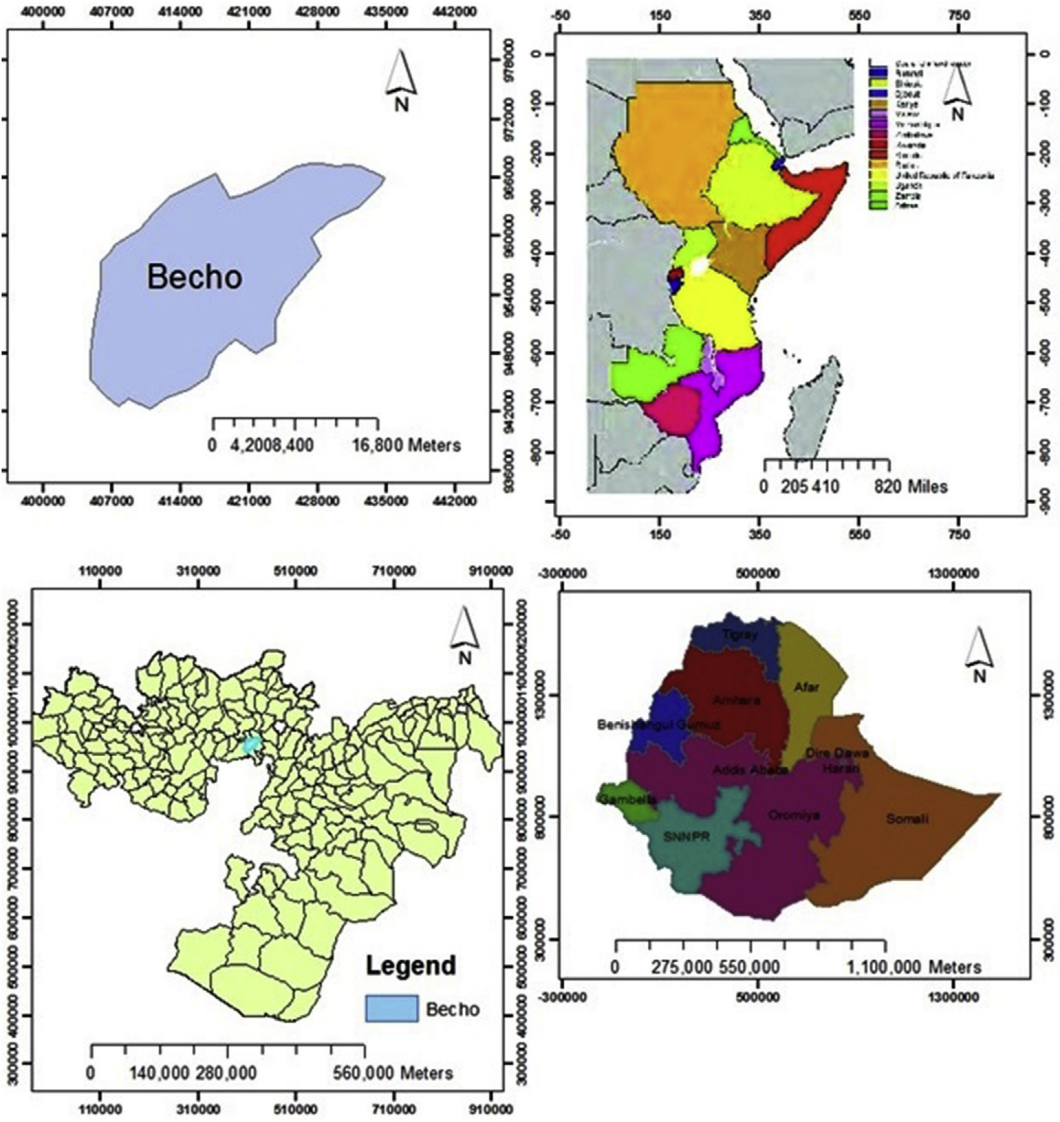
Map of Becho Woreda (district).

The topography of the district is generally plain with undulating and hilly land. Vertisol (black soil), which is moderately fertile, is the main soil type in the district, accounting for about 85% of the soils. This is followed by red soils (10%), and the remaining areas comprise of other types of soils. Total land area of the woreda is roughly 44,775 hectares (ha), of which 32,432 hectares can be cultivated, and 587 hectares can be considered uncultivable (Tolossa et al., 2015; Elias, 2016). Mixed farming (crop and livestock production) is the dominant livelihood of the rural residents. Major crops produced in the district are tef, wheat, and chickpea. Tef and wheat are grown from July to November whereas chickpea is grown from September to December (CASCAPE, 2015; Elias and van Beek, 2015) (see Figure 1).

### 2.2 Sampling technique and data collection instruments

Becho district is selected as the study site due to its agroecological suitability to wheat-chickpea double cropping, and the double cropping intervention carried out by CASCAPE project in the last 8 years. A two-stage stratified random sampling method was employed to draw samples. The study categorized the survey population at two levels, namely at the rural Kebele level and at the farm household level. In the first stage, rural Kebele administrations were stratified into two categories as potential and less potential wheat and chickpea growers. Accordingly, among the numerous Vertisol dominant Kebeles in the district, four potential wheat and chickpea producing Kebeles were selected using simple random sampling. In the second stage, households from each Kebele were stratified into two groups based on their adoption status as adopters and non-adopters of wheat-chickpea double cropping, and sample households were selected randomly from each Kebele.

The sample size (sample households) was determined based on Yamane's (1967) formula described below.

$$n=\frac{N}{1+N{\left(e\right)}^{2}}$$

Where *n* is the sample size (household), *N* is the total household in the district, and *e* is the level of precision. As the unit of analysis for this study was at household level, 7% precision level was considered in light of increasing homogeneity within adoption stratum and heterogeneity between strata (Kebeles). Substituting the value into the above formula provided a sample size of 203 households. The sample households were taken proportionally from each of the four Kebeles (strata). The number of respondents in each rural Kebele is shown in Table 1 below.

**Table 1:** Proportional sampling of the four Kebeles (strata).

Strata	Name of the Kebele	Population = N (Household)	Proportional sample (Household)
Kebele-1	Awash Bune	1023	77
Kebele-2	Soyama	673	51
Kebele-3	Baballi	530	40
Kebele-4	Batu	459	35
Total		2685	203

Structured questionnaire was administered to collect data from the sample households. This tool was used in particular to collect socioeconomic and agriculture related data from the sample households. The questionnaire was translated into local language (Oromo language) and was pre-tested. The data collection was carried out with full consent of the respondents. In addition to the quantitative data, qualitative data were also collected from a focus group discussion. One focus group discussion among eight farmers (two from each Kebele; one model and one non-model farmer) was organized to enrich and cross-check the data obtained from the questionnaire.

### 2.3 Method of data analysis

Descriptive statistics (frequency and percentage) and propensity scoring matching (PSM) were used to analyze the data with due emphasis on PSM. In descriptive analysis, statistical tests such as t-test and chi^2^ were used in assessing the relationship between the dependent variable and the explanatory variables.

#### 2.3.1 Propensity score matching (PSM)

The purpose of the study is mainly to estimate the impact of adopting wheat-chickpea double cropping technology on crop productivity and income of adopters in comparison to those households who are non-adopters. In reality, farmers were not randomly assigned into the treatment and control groups. Thus, the probability of a given farm household to fall into a treatment or control category depends, among other factors, on personal and farm characteristics of the household. Given this scenario, it is crucial to take care of the potential selectivity bias in the samples. Data often do not come from randomized trials but from non-randomized (observational data), which is the case in this study. Propensity score matching method helps to control for such potential selection bias. Rosenbaum and Rubin (1983) proposed propensity score matching as a method to reduce the bias in the estimation of treatment effects with observational data sets. Since this study was conducted on cross-sectional survey data, which reflects non-randomized data setting (control and treated groups), PSM provides the chance of constructing artificial control groups by matching each treated unit (adopters) with a non-treated unit (non-adopters) of similar characteristics. Because of this advantage, PSM was chosen to analyze the data.

The main purpose of using the PSM method is to find a group of non-treated farmers (those who did not adopt the double cropping technology) similar to the treated groups (adopters of the double cropping technology) in all relevant observable characteristics with the only difference being one group adopts and the other group being non-adopters. That is to say, with matching methods, one tries to develop a counterfactual or control group which is similar to the treatment group in terms of observed characteristics. Each participant/treated group/is matched with observationally similar non-participant/control or comparison group/and then, the average difference in outcome across the two groups is compared to get the program treatment effect.

The propensity score matching approach captures the effects of different observed covariates on participation in a single propensity score or index. Then, outcomes of participant and non-participant households with similar propensity scores/common support/are compared to obtain the program effect. Households with no match are dropped/off support/because no basis exists for comparison. By doing so, it is possible to identify welfare effect of adopting double cropping technology on the outcome of interest (yield per hectare and income) by the following equation:

(1) $$E\left[Y_{1}-Y_{0}|D=1\right]=E\left[Y_{1}|D=1\right]-E\left[Y_{0}|D=1\right]$$

Where, Y is yield per hectare or farm income and D takes the value 1 for adopters (treatment group) and 0 for non-adopters (control group). Thus, the outcome of interest is the average difference in $Y_{1}$ and $Y_{0}$. However, this matching exercise tries to estimate only; ***E*[*Y***_***0***_ ***|D =* 1]**, which is the counterfactual or the unobservable case, since one farmer falls only in one state (either in the treatment group or in the control group) at a time. In our case, this means, trying to estimate the impact of being an adopter on yield per hectare and farm income for those farmers who are actually in the control group.

For experimental data in which farmers are randomly assigned to the treatment and control groups, it would have been possible to estimate the average treatment effect (ATE) as:

(2) $$\mathrm{ATE}=E\left[Y_{1}\left|D=1\right]-E\left[Y_{0}\right|D=0\right]$$

However, this study relied only on observational data. Hence, instead of ATE, the issue of interest for this study is average treatment effect on the treated (ATT). Thus, following Rosenbaum and Rubin (1983), the equation below was applied to solve the selection bias:

(3) $$E\left[Y_{1}-Y_{0}|Z,D=1\right]=E\left[Y_{1}|Z,D=1\right]-E\left[Y_{0}|Z,D=1\right]$$

Where, Z is a set of covariates which determine the adoption status of farmers. If the probability of being an adopter is determined by Z, then it is possible to establish a control group of non-adaptors that are similar in Z relative to adopters (the treatment group). Thus, from Eq. (3), it is possible to estimate the average treatment effect on the treated (ATT) as:

(4) $$ATT=E\left[Y_{1}-Y_{0}|P\left(Z\right),D=1\right]=E\left[Y_{1}|P\left(Z\right),D=1\right]-E\left[Y_{0}|P\left(Z\right),D=0\right]$$

Where, P(Z) is the probability of selection conditional on Z or it is the propensity score (Pscore) which is P(Z)≡ Pr (D = 1 |Z).

Hence, the matching was done after the propensity scores (P-scores) were calculated. Calculating the propensity score is crucial since it is difficult to do the matching on each explanatory variable when there are many covariates. The main purpose of the propensity score estimation is to balance the observed distribution of covariates across the two groups. Matching test was also conducted after matching to check whether or not the differences in covariates in the two groups in the matched sample have been eliminated.

After matching, sensitivity analysis was performed as a final diagnostics to check the sensitivity of the estimated treatment effect to small changes in the specification of the propensity score (Dehejia and Wahba, 2002). Sensitivity analysis is recommended to assess the sensitivity of results to uncontrolled confounding variables (Rosenbaum, 2002). Matching estimators work under the assumption that a convincing source of exogenous variation of treatment assignment does not exist. Based on this principle, sensitivity analysis was run to check whether unobserved covariates have effect on the results by creating biases or not. Furthermore, after ATT is calculated, it is vital to test whether the estimated ATT results are effective or not. Thus, sensitivity analysis was undertaken to detect the identification of conditional independence assumption (CIA) if it was affected by the confounder or not.

#### 2.3.2 Outcome indicators and explanatory variables

The outcome variables (yield and income) follow from the adoption of wheat-chickpea double cropping, and thus adoption is the dependent variable and it is dichotomous. Thus, adoption has two values, i.e. 1 and 0, where ‘1’ stands for households who have adopted wheat-chickpea double cropping technology, while ‘0’ denotes non-adopters. On the other hand, yield (productivity) and farm income are outcome variables. Yield is the first outcome variable which measures production per hectare. It is a continuous outcome variable measured in terms of kilogram/hectare (kg/ha). Although wheat and chickpea are commodities of interest in the double cropping study, yield advantage comparison was made on the wheat crop that both adopters and non-adopters had cultivated.

Farm income is the second outcome variable which measures the total amount of annual farm income earned by farm households. It is continuous variable measured in terms of Ethiopian Birr/Euro (*Based on commercial Bank of Ethiopia's exchange rate, May 2019*; *1 Euro = 32 Birr)*. The farm income was obtained from both production of wheat and chickpea for the treated (since they adopted wheat and chickpea double cropping) and only wheat for the controls (since they are mono croppers or non-adopters).

As stated above, PSM is dependent on matching control and treatment groups in terms of observed characteristics or covariates which determine the adoption status of farmers. In this regard, seventeen variables that are assumed to affect adoption of wheat and chickpea double cropping technology were considered based on theoretical and empirical literature in similar studies, and are listed in Table 2 below.

**Table 2:** Summary of explanatory variables.

Name of Variable	Description	Type	Unit of measurement
Sex	Sex of the household (HH) head	Dummy	1 = Male; 0 = Female
Age	Age of the HH head	Continuous	Year
Education	Literacy status of the HH head/Able to read & write/	Dummy	1 = Yes (able to read and write); 0 = Otherwise
Farmer type	Type of farmer (HH head)	Dummy	1 = Model; 0 = Non-model As categorized by the District's Office of Agriculture
Family size	Total family size	Continuous	Number
Labor	Labor availability (hired or household labor)	Dummy	1 = Yes; 0 = No
Farm size	Farm size owned by the HH	Continuous	Hectare
Non-farm income	HH's status in getting non-farm income	Dummy	1 = Yes; 0 = No
Livestock holding	Livestock holding of the HH	Continuous	TLU
Training attendance	Training attendance on double cropping	Dummy	1 = Yes; 0 = Not taken
Access to improved seeds	Access to improved seeds	Dummy	1 = Have access; 0 = No access
Access to BBM	Access to broad bed maker (BBM)	Dummy	1 = Have access; 0 = Otherwise
Fertilizer Access	Access to fertilizer	Categorical	1 = Yes (there is always access); 2 = Medium (it is not always accessible); 3. No access (not at all)
Market Access	Access to the nearest market	Dummy	1 = Have access; 0 = Otherwise
Credit Access	Access to credit	Categorical	1 = Yes (there is always access); 2 = Medium (it is not always accessible); 3 = Have enough money and no credit need; 4 = No (not access at all)
Bio-fertilizer	Access to Bio fertilizer	Dummy	1 = Yes; 0 = Otherwise
Extension contact	Frequency of extension contact per cropping season days per week	Continuous	Days

## 3 Results and discussion

### 3.1 General characteristics of the households

As indicated in Tables 3 and 4 below, from the entire two hundred and three (203) sample farmer households interviewed, 189 (93.1%) were male headed and 14 (6.9%) were female headed households. With regards to education status (ability to read and write), the chi-square test result showed the presence of significant relationship between education and adoption at 1% confidence level. For farmer type as categorized by the district's Office of Agriculture as model (“A & B” level farmers) and non-model (“C” level farmers); there is significant association (dependence) between adoption and farmer type (model and non-model category) at 1% significance level. It is found that larger proportions of adopters are model farmers. The chi-square test for labor availability showed that, there is significant association at 1% level between labor and adoption of the double cropping. Similarly, there is significant association (dependence) between adoption and training. As could be seen in Table 3 below, those who had training in double cropping are found to be more adopters than the untrained ones.

**Table 3:** General characteristics of sample households (categorical variables).

Variables		Adopters (N = 83)		Non- adopters (N = 120)		Total (N = 203)		
		No.	%	No.	%	*x*^2^-test	No.	(%)
Gender/Sex/	Male	78	94.0	111	92.5	0.17	189	93.1
	Female	5	6.0	9	7.5		14	6.9
Education	Yes	58	69.9	31	25.8	38.66	89	43.8
	No	25	30.1	89	74.2		114	56.2
Farmer type	Model	42	50.6	7	5.8	53.7	49	24.1
	Non-model	41	49.4	113	94.2		154	75.9
Labor	Yes	66	79.5	73	60.8	7.935	139	68.5
	No	47	39.2	17	20.5		64	31.5
Training given	Yes	79	95.2	26	21.7	106.18	105	51.7
	No	4	4.8	94	78.3		98	48.3
Access to improved seeds	Yes	74	89.2	12	10.0	126.07	43	21.2
	No	9	10.8	108	90.0		68	33.5
Access to fertilizer	Yes	82	98.8	117	97.5	1.46	199	98.0
	Medium	1	1.2	1	1.7		2	1.0
	No	0	0.0	2	1.7		2	1.0
Access to BBM	Yes	76	91.6	19	15.8	113.026	95	46.8
	No	7	8.4	101	84.2		108	53.2
Access to market	Yes	68	81.9	40	33.3	46.53	108	53.2
	No	15	18.1	80	66.7		95	46.8
Access to credit	Yes	63	75.9	101	84.2	3.05	164	80.8
	Medium	8	9.6	5	4.2		13	6.4
	No need for credit	3	3.6	4	3.3		7	3.4
	No	9	10.8	10	8.3		19	9.4

**Table 4:** General characteristics of sample households (continuous variables).

Variables	Adopter (N = 83)		Non-adopters (N = 120)		t-test
	mean	Std	Mean	Std	
Age	44.5	9.23	42.2	10.71	1.57
Family size	6.89	1.96	6.14	2.22	2.47 ∗∗
Owned land holding (ha)	2.73	1.25	2.18	1.08	3.35 ∗∗∗
Livestock holding (TLU)	6.23	4.469	3.98	2.79	4.39∗∗∗
Non-farm income (Birr)	7645.8	10109.69	10239.3	7920.75	1.0053

∗∗∗ 1% significance level, ∗∗ 5% significance level (Source: Own survey result, 2019).

Access to improved seeds of wheat and chickpea is also found to show strong significant association with adoption at 1% significance level. Adopters have better access to improved seeds while larger proportions of non-adopters have very less access to improved seed. With regard to access to broad bed maker (BBM), the chi-square test shows a significant association between adoption and access to BBM at 1% confidence level. The chi-square test for access to market also shows a significant association between adoption and access to market at 5% significance level.

As regard to family size, the total sample households have averagely about 6 family members with a minimum of 1 and a maximum of 13. The average family size of the adopters was nearly 7 people, while it was about 6 persons for non-adopters. The t-test distribution of family size between adopters and non-adopters is significant at 5% significance level, and this shows a statistically significant mean difference between family size of adopters and non-adopters.

As a major physical wealth of rural societies, cultivable farmland possession among the households ranged from the smallest of 0.5 ha to the highest 7.0 ha. The t-test distribution of owned farm size between adopters and non-adopters is significant at 1% level, and adopters’ average owned farm size (2.73 ha) is bigger than that of the non-adopter households (2.18 ha). Similarly, the t-test distribution for livestock holding (measured in tropical livestock unit/TLU) between adopters and non-adopters is significant at 1% level. While adopters possess an average of 6.23 units of TLU, the non-adopters holding is only 3.98.

### 3.2 Propensity score distribution, matching and balancing quality

Before doing the actual matching, likelihood of adoption for all adopters/treated 83 (40.89%) and non-adopters/controlled 120 (59.11%) was carried out. Matching of treated and control households was then carried out to determine the common support region. The basic criterion for determining the common support region is to discard all off-support observations whose propensity score is smaller than the minimum propensity scores of adopters (treated) and larger than the maximum of the (control group) non-adopters. This leads to the exclusion of all observations out of the overlapping region. A common support condition was imposed based on the propensity score distributions of the households with and without the program (adoption of wheat-chickpea double cropping). The common support option was selected and the balancing property is found to be satisfying in matching individuals with similar observable characteristics with the treated group.

Thus, the common support assumption is satisfied in the region of [0.0009342, 1] for sample households. This means that households with estimated propensity scores less than “0.0009342” and greater than “1” were not considered in the matching undertakings. As a result of this restriction, a total of 190 sample households (83 treated and 107 controls) were identified for the estimation process, whereas 13 sample households (all from the control sample households) were discarded from the total 203 observations (Table 5).

**Table 5:** Blocks of propensity score and common support.

Blocks of p-score	Adoption status		Total
	Non adopter	Adopter	
0.0015934	90	2	92
0.2	7	2	9
0.4	4	6	10
0.6	1	8	9
0.8	5	65	70
Total	107	83	190

- The common support option has been selected.

- The balancing property is satisfied.

After matching was carried out, quality of balance between the observable covariates was checked. The main aim of the propensity score matching is to balance the covariates between the groups. Thus, the checking was carried out considering the standardized percentage bias and mean bias before and after matching (Rosenbaum and Rubin, 1983). For good matching performance, the bias should be less than 5%, Ps R^2^ should be very close to zero and t-test and p-value (p > chi2) need to be non-significant after matching. Accordingly, specifications with various covariates that showed poor matching quality (mean bias and the other testing measure) were discarded till the good performance level was achieved. After matching, the average mean bias is 2.7, which is much less than 5 (before matching, it was 119.2) and P value is non-significant (0.996). Besides, Ps R^2^ is very close to zero (0.002). With these results, the balancing quality is achieved (Table 6) paving the way to the analysis of average treatment effect (ATT). Average treatment effect result is reliable only after balancing quality is achieved (Rosenbaum and Rubin, 1983).

**Table 6:** Summary output of matching quality.

Sample	Ps R2	LR chi2	p > chi2	Mean Bias	Med Bias	B	R
Unmatched	0.675	185.38	0.000	119.2	112.4	318.9	0.49
Matched	0.002	0.38	0.996	2.7	3.7	9.5	1.08

After obtaining good matching quality as shown in Table 6, the average treatment effect on the treated (ATT) was estimated in the second stage. Robustness of the PS match2's ATT result was also checked by running matching algorithms such as the nearest neighbor (NN), kernel and stratification matching techniques.

### 3.3 Choice of matching algorithms

There are a number of matching algorithms. The most commonly used matching algorithms are nearest neighbor (NN), kernel-based, and stratification matching methods. To check robustness of the results and select the best, these matching methods were employed for both of the outcome variables. In general, all the four matching methods revealed that adopters of wheat-chickpea double cropping have generated significantly higher output (wheat yield and farm income) as compared to the non-adopters. Psmatch2 result of both outcome variables was selected as best ATT in comparison to the other matching algorithms since Psmatch2 result represented much larger matched sample or common support (Table 7).

**Table 7:** Performance of matching estimators for sample households for wheat yield and farm income.

A. Performance of matching estimators for sample households for wheat yield				
Matching estimator	Matched sample	ATT (yield (kg/ha)	Bootstrapped Std. err	t-stat
Nearest neighbor matching method	134	927	2.19	4.21
Kernel matching method	155	930	1.18	7.9
Stratification method	155	920	1.45	6.35
Psmatch2	190	696	2.01	3.46
B. Performance of matching estimators for sample households for farm income in Ethiopian Birr (ETB) or in Euro.				
Matching estimator	Matched sample	ATT-income in ETB and Euro	Bootstrapped Std. err	t-stat
Nearest neighbor matching method	134	22,064.76 ETB or 689.52 Euro	2,304.1	9.57
Kernel matching method	155	22,715.45 ETB or 709.85 Euro	1,961.7	11.5
Stratification method	155	22,681.6 ETB or 708.8 Euro	2,030.7	11.1
Psmatch2	190	18,564 ETB or 580.125 Euro	3,475.9	5.34

### 3.4 Sensitivity analysis results

As could be seen in Table 8 below, the sensitivity analysis result shows that the significance level of ATT is unaffected and is insensitive to external change. Therefore, the Conditional Independence Assumption (CIA) remained significant and the results were not sensitive to confounders and this indicates absence of external cofounders (variables) which affect the results calculated for ATT of both outcome variables. As depicted in Table 8, the Gamma values from 1 to 2 have upper significance level of 9.8e-09 and lower significance level 0 for wheat yield; 7.9e-14 and lower significance level 0 for farm income. This result indicates that the technical efficiency is significant or robust and the ATT results of both outcome variables were not sensitive to confounders.

**Table 8:** Sensitivity analysis for outcome variables.

a. Yield (kg/ha), 2018 cropping season			b. Farm income		
Gamma	σ^+^ (sig^+^)	σ^-^ (Sig^-^)	Gamma	σ^+^ (sig^+^)	σ^-^ (Sig^-^)
1	3.3e-15	3.3e-15	1	1.2e-15	1.1e-15
1.05	1.5e-14	6.7e-16	1.05	5.7e-15	2.2e-16
1.1	5.7e-14	1.1e-16	1.1	2.3e-14	0
1.15	2.0e-13	0	1.15	7.9e-14	0
1.2	6.1e-13	0	1.2	2.5e13	0
1.25	1.7e-12	0	1.25	7.3e-13	0
1.3	4.6e-12	0	1.3	2.0e-12	0
1.35	1.1e-11	0	1.35	4.8e-12	0
1.4	2.6e-11	0	1.4	1.1e-11	0
1.45	5.6e-11	0	1.45	2.5e-11	0
1.5	1.2e-10	0	1.5	5.2e-11	0
1.55	2.3e-10	0	1.55	1.0e-10	0
1.6	4.3e10	0	1.6	2.0e-10	0
1.65	7.8e-10	0	1.65	3.6e-10	0
1.7	1.4e-09	0	1.7	6.4e-10	0
1.75	2.3e-09	0	1.75	1.1e-09	0
1.8	3.9e-09	0	1.8	1.8e-09	0
1.85	6.2e-09	0	1.85	3.0e-09	0
1.9	9.8e-09	0	1.9	4.7e-09	0
1.95	1.5e-08	0	1.95	7.3e09	0
2	2.3e-08	0	2	1.1e-08	0

∗gamma, log odds of differential assignment due to unobserved factors; sig^+^, upper bound significance level; and sig^-^, lower bound significance level.

### 3.5 Impact of adoption on grain yield

It has to be noted that wheat is the common crop for both the treated and the controls to estimate yield based ATT. The estimated average treatment effect (ATT) of sample households shows a significant effect of wheat-chickpea double cropping on wheat yield of treated groups of smallholder farmers, i.e., adoption of wheat-chickpea double cropping generated positive and statistically significant wheat yield difference as compared to that of non-adopters. As shown in Table 9 below, the average treatment effect on the treated (ATT) of wheat yield of adopters and non-adopters for the 2018 production season has a yield difference of 700 kg/ha in favor of the adopters/treated group at statistically significant level of T-stat 3.46. This shows that adopters have recorded a yield advantage of 49.3% over the non-adopters. In terms of the average yield (kg/ha), adopter group of farmers harvested an average wheat yield of 2,120 kg/ha, while the non-treated groups harvested an average wheat yield of 1,420 kg/ha. This result indicates that wheat-chickpea double cropping is worth to adopt. Adopter participants in the focus group discussion indicated that the practice of double cropping is very useful in improving soil fertility of their land, increasing production and productivity, and at the same time it is improving their income and livelihoods in general. A study conducted by Eshete et al. (2017) confirm the same where they stated double cropping enhancing water and nutrients’ distribution in the soil, which in turn, contributed to yield increment.

**Table 9:** Average Treatment Effect on Treated (ATT) for yield (kg/ha).

Outcome variable	Sample	Treated	Control	Difference	Difference in percentage	S.E.	T-stat
Yield (wheat) (kg/ha)	Unmatched	2,311.44	1,652.5	658.9	39.88	0.7786	8.46
	ATT	2,120	1,420	700	49.30	2.011	3.46

### 3.6 Impact of adoption on farm income

As observed in the case of the yield impact, the estimated average treatment effect (ATT) of sample households depicts that adoption of wheat-chickpea double cropping has created a significant average positive farm income difference between adopters (wheat and chickpea double croppers) and non-adopters (wheat mono croppers) for the 2018 cropping season. As shown in Table 10 below, the treated group of farmers earned an average annual income of 22,692.8 Birr (709.15 Euro) from sale of both wheat and chickpea as adopters, while the non-adopters/control groups earned an average annual income of 4,128.5 Birr (129 Euro) as mono croppers from the sale of wheat only, and the result is statistically significant at T = 5.34. This shows that treated group of farmers have an advantage of 18,564 Birr (580.125 Euro) over the control group, and the percentage change is 449.65% over the non-adopters. Alike the yield advantage, the treated (adopters) are beneficiaries of economic advantage as a result of adoption of wheat-chickpea double cropping. Both the yield and income result favored the treated groups who are adopters of the wheat-chickpea double cropping.

**Table 10:** Average Treatment Effect on Treated (ATT) for farm income (Birr/Euro per annum).

Outcome variable	Sample	Adopters	Non-Adopters	Difference	Difference in percentage	S.E.	T-stat
Farm income *(*wheat and chickpea*)* in Ethiopian Birr and Euro/Annum	Unmatched	26,354.04	5,087.5	21,266.5	418.02	1,513.32	14.05
	ATT	22,692.8 ETB/709.15 Euro	4,128.57 ETB/129 Euro	18,564.2 ETB/580.125 Euro	449.65	3,475.91	5.34

Based on the above results on yield and income, one can see that non-adopters are in disadvantageous position on two grounds: i) Adopters have better yield advantage on wheat itself with 696 kg/ha difference over the non-adopters just by adopting the double cropping system. ii) Adopters practice double cropping by immediately planting chickpea on residual moisture after harvesting of wheat on that same plot. The price of chickpea is higher than wheat. As per the local market price during the survey data collection time, farmers indicated that wheat was being sold for 1,000–1,200 Birr/100kg or 31.25–37.5 Euro/100kg. On the other hand, local market price of chickpea was 1,400–1,900 Birr/100kg or 43.75–59.375 Euro/100kg. Since adopters harvest chickpea in addition to wheat in the same season, on the same plot, they are better off than the non-adopters (keeping other farm income constant) in generating additional income. In such a way, one can clearly note how adopters managed to earn an average treatment effect of 18,564 Birr or 580.125 Euro difference over the non-adopters.

## 4 Conclusion

This study was conducted to evaluate the impact of wheat-chickpea double cropping among smallholder farmers in Ethiopia. The study considered adopters and non-adopters of double cropping so as to compare their differences in productivity and income obtained from their farming directly linked to wheat and chickpea. Accordingly, the study revealed that adoption of wheat-chickpea double cropping has a positive and significant impact both on yield and farm income of smallholder farmers. The adoption decision of households for wheat-chickpea double cropping has generated a yield difference of 696 kg/ha as compare to that of non-adopters. In terms of the average yield, the treated group of farmers harvested an average wheat yield of 2,120 kg/ha, while the non-treated groups harvested an average wheat yield of 1,420 kg/ha. Similarly, the treated group of farmers earned an average annual income of 22,692.8 Birr or 709.125 Euro from sale of both wheat and chickpea as adopters, while the control groups earned an average annual income of 4,128.5 Birr or 129 Euro as mono croppers from the sale of wheat only. Averagely, the treated groups of farmers have an advantage of 18,564 Birr or 580.125 Euro over the control group.

These results imply that scaling out of wheat-chickpea double cropping can contribute to food security and rural livelihood improvement through increment of productivity and farm income. Hence, encouraging farmers towards adoption of wheat-chickpea double cropping is vital by properly identifying and addressing enablers, drivers, and hindering factors of adoption and scaling up, and the study revealed that access to improved seeds, training on double cropping, involvement in non-farm income activities, access to broad bed maker and access to fertilizer should be adequately addressed.

## Declarations

### Author contribution statement

Desalegn Haileyesus: Conceived and designed the experiments; Performed the experiments; Analyzed and interpreted the data; Contributed reagents, materials, analysis tools or data; Wrote the paper.

Abate Mekuriaw: Conceived and designed the experiments; Analyzed and interpreted the data; Contributed reagents, materials, analysis tools or data; Wrote the paper.

### Funding statement

This work was supported by BENEFIT-CASCAPE (The Bilateral Ethiopian-Netherlans Effort for Food, Income and trade Partnership - Capacity Building for Scaling Up of Evidence Based Best Practices in Agricultural Production in Ethiopia).

### Data availability statement

The data that has been used is confidential.

### Declaration of interests statement

The authors declare no conflict of interest.

### Additional information

No additional information is available for this paper.

## Footnotes

1 CASCAPE (Capacity Building for Scaling Up of Evidence Based Best Practices in Agricultural Production in Ethiopia) project is funded by the Netherlands government and implemented by five Universities in Ethiopia and Wageningen University, the Netherlands. The aim of the project is to support the Ethiopian government to increase agricultural productivity by verifying and testing best practices and innovations in agricultural production and identifying the circumstances under which farmers are prepared to take up innovations, and scale up these best practices to the wider farming communities. Addis Ababa University implemented the project in the Central Highlands of Ethiopia.
